# Surgery, Medicine, and a Little Logic*Read before the Brooklyn Hahnemannian Union, January, 1897.

**Published:** 1898-10

**Authors:** B. Fincke

**Affiliations:** Brooklyn, N. Y.


### SURGERY, MEDICINE, AND A LITTLE LOGIC.*

B. FINCKE, M. D., BROOKLYN, N. Y.

The late celebrated surgeon, Bilbroth, declared that medicine must become more and more surgical. He is no more. It is not known that he died from a surgical operation, so he must have died of some disease which was not amenable to surgery. He was then in the prime of his life, and therefore serves as a warning example against his own dictum, for if there had been any operation in surgery to help him it should have saved him. But he died in spite of the eminent surgical attainments of which he was a shining light, and was a victim to that conception of medicine which was not surgical enough. If his case was not a surgical one it would point to a deficiency in the internal medical treatment which was not medicinal—*i. e.*, not healing enough. His prediction, however, has been verified in the few years following, since medicine indeed has been made more and more surgical, so that nowadays surgeons are not afraid to cut for any ailment flesh is heir to. Armed with their anæsthetics, antiseptics, and disinfectants, they are not appalled by any ever so serious inroad into the organism. If the operation succeeds, there is no end of praise. If not, nobody finds fault with the operator. Their great point is the pathological diagnosis. Whether it be correct or not, is of no account, because at any rate the knife will reveal the truth. If it is erroneous, the wounds inflicted are soon healed up under their skillful hands, and nobody is the wiser. The practice of surgery is more lucrative than that of medicine. It has the appearance of bringing the ailment to a prompt termination, but the time following the operation is not counted to be as tedious as it would have been deemed if under homœopathic treatment. The people would rather submit to a daring operation than to trust a careful and no less skillful management of the medical man. Mistakes are fre-

---

* Read before the Brooklyn Hahnemannian Union, January, 1897.

quently made, but reputation and professional connections protect those who make them. Operations are made without any hope of recovery, cutting short any chances which may be offered by medicinal treatment of the right sort. The public is kept in ignorance and browbeaten by the assumed wisdom of the surgeon eager to operate. The patients are carried to the hospitals and laid on the operating table without knowing what is to become of them. Relatives interested in the success of the operation are kept out to prevent their interfering with the surgeons, and are apprised of the condition of the patient only when they have to carry him out again dead. The surgeons have acquired this great power over the public, which admires them as much as it fears them, and find their stronghold mainly in the sanitary department, which curtails the rights of the medical men more and more, by viewing disease as having a material origin and requiring material remedies—among them compulsory measures. There is no remedy for this, because these men have the ear of the political and social powers in the communities, using their legislative, judiciary, and executive organs for their self-interests. Statistics, properly managed, is the powerful weapon with which to make the people do their bidding. Falsifying the records is common practice. Like that great political thief who stole millions from the commonwealth by his corrupt practices, they ask sneeringly, "What are you going to do about it?" It is almost impossible to kill that boisterous allœopathic Goliath, and yet the Hahnemannian with his little pebble will be the David to slay him in time, if the disciples of the great reformer of medicine do not swerve from his doctrine in its entirety, and do not compromise with the enemy of the healing art and science.

The great danger for medicine from surgery comes from its arrogance, which teaches them to consider every medical case as belonging to their peculiar domain. In Hahnemann's time surgery was still in its infancy, and yet in his *Organon*, § 186, he limits surgery wisely to the removal of the external impediments to the healing to be expected from the life-force alone. If, in addition, he deems it proper, as among the justifiable measures of surgery, to open a cavity of the body in order to take out a molesting substance, or to procure an exit for extravasated or accumulated fluids, he is not on that account to be hailed as the father of laparotomy and the numerous operations nowadays executed under that name. For the Cæsarian section traces its history to the Dark Ages of the ancients, and laparotomy dates from the sixteenth century. The construction of the surgical paragraph in *The Organon* shows Hahnemann by no means in the light of a Billroth, as has been intimated by one of our young surgeons, but limits it to the cases where the life-force, assisted by proper homœopathic medication, cannot overcome the conditions of disease, and needs the wisely-conducted hand of the surgeon to save the organism from destruction. But it should never be applied before this necessity arises, and an operation should never be performed which severs the life-thread without gaining another end than the success of the operation producing a surgical death. There is a necessary antagonism between the surgeon and physician which is most difficult to reconcile in the medical man who is expected to be both. While medicine tries to preserve the organism and its parts by remedies, which should be so prepared that they could never jeopardize the life of the individual, and bring about the health of the patient with the least expenditure of force, the surgeon must sacrifice parts of the organism by mechanical force, which, indeed always should be also the least possible, but must always be a more or less dangerous proceeding. as it severs the continuity of the constituents of the organism, and causes loss of blood and strength by forcible means. It stands, therefore, to reason that no operation should be performed as long as there is a chance for the organism to recover under proper homœopathic treatment, with remedies standing, in their action, on the same plane as the life-force, and in this respect similar. Since Hahnemann's invention of the method of preparing medicines by potentiation, the armamentarium of the physician has been enriched with more powerful means for healing than ever before have been known. It is, therefore, a sacred duty for the surgeon, as well as for the physician, to make himself familiar with this subject in order to let his patients have the benefit of it.

Tumors, large and small, in and outside of the body, have been removed by the use of high potencies, effusions in cavities have been discharged under their influence, through the legitimate channels of the system. Abscesses of all kinds opened spontaneously and healed up without using the knife, through their instrumentality. Blood-poisoning and septicæmia have been prevented in the same manner, and some of our best men who have wounded themselves in operating, and given poison access to their systems, have been saved by Homœopathy, whilst others had to succumb under the insufficiency of surgical allœopathy, not knowing of the powerful Hahnemannian aid. Poisoning by the bite of venomous snakes has been cured by the homœopathic high potency as well as the dreadful and mysterious hydrophobia. These are all matters of record, and well known to the Hahnemannian branch of the homœopathic world.

If it is not considered a crime to operate without sufficient cause in the sense acknowledged by the laws of the commonwealth, it is a crime nevertheless in the sense of science and humanity, which, if it goes unpunished in this world, still waits for its unfailing judge in the one beyond.

The highest authority in the organism, in the mind of the physico-chemical school of the present day, is the blood, to which their efforts of curing are mainly directed. The nervous system is only appealed to for benumbing or extinguishing pain by palliatives and anæsthetics. This school will have to discover again the, by her, despised life-force, without which no organism is to be thought of; that great authority which holds all the organs and their functions in the body in control, by directing the system of the highest dignity in the organism, viz.: the brain and the nervous system attached to it, in an intelligent manner, as yet little understood by even the wisest of this generation. In any injury by accident this ever-present authority is only vaguely acknowledged by estimating the shock which it may exert upon it. Hahnemann had a clear perception on that point when in his *Chronic Diseases* I, p. 43, he says: "If the small-pox or cow-pox catches it is done in the moment when, by the inoculation of the same, the morbid fluid in the bloody scratch comes in contact with the nerve lying open, which then, in the same moment, communicates the disease dynamically and irrevocably to the life-force (the whole nervous system)." In like manner "the infection with miasms of the acute as well as of the chronic diseases happens, no doubt, in one single moment —*i. e.*, in the one favorable to the infection." To this should be added the bites of venomous and rabid animals. The impression made upon the sensitive nerve precedes the blood-poisoning, as the symptoms denoting the change of the system require more or less time to develop. The symptoms show the reaction of the life-force upon the intoxication, and reaction follows action in time.

Let us here stop a moment to express our admiration of the wisdom of our master who pronounced this great truth, which, after the lapse of seventy years, condemns the lately broached doctrine that all diseases start from bacteria, microbes, bacilli, cocci, etc., and leads to remedial measures which sacrifice still more lives than have been victimized before. Who can help acknowledging the truth of Hahnemann's observation when looking at the phenomena of natural and accidental pathogenesis by infection, incision, and inoculation? Take, for example, that mysterious disease called, not quite properly, hydrophobia, viz.: Lyssa. No hydrophobic bacterium has so far been detected, and none probably will be. Even if it should it would no more confirm the origin of this disease by the parasite than the origin of vaccinal disease after inoculation. Even the most advanced methods of chemistry combined with microscopy, to which spectroscopy and photography must be added, cannot detect the least deleterious thing in the saliva of the mad dog. The blood is not changed characteristically by it, but all the symptoms point to a severe affection of the nervous system, through which the life-force reacts upon the organs, presenting the peculiar symptoms in order to ward off the danger of destruction. No sedative will ever do any good in such a state. It will only turn the narcosis into its opposite and render the case more formidable and incurable. Nor will the local application of cautery and burning be of any account. Only the homœopathic remedy will be useful, as we know from Hahnemann, and from Bœnninghausen's well authenticated case which he cured with a dose of Hyoscyamus-niger, 12th cent. potency. Could such a fine dose, which borders on infinitesimality, have acted chemically? The physico-chemical school asserts that blood-poisoning had taken place and hence must be met by remedies which enter into the circulation immediately—*i. e.*, by inoculation. They forget the primary injury which they inflict on the nerve "lying open" in their operation. If there were any curative action, which is doubtful in most cases, it could only be by an infinitesimal of the nosode touching the wounded nerve, thus communicating the homœopathic healing impulse to the life-force (the whole nervous system), and in its action healing by equalization of the disturbed state of the life-force. Nothing is introduced directly into the circulation of the blood in that beautiful method of healing hydrophobia by steam or the vapor bath; nor in the dry method of producing profuse perspiration by submitting the body to the heat of an alcohol lamp in the closed cabinet; nor by the primitive method of confining the patient in feather beds till he ceases to struggle, when he is found, on freeing him, to be cured by the profuse perspiration excited. We have the extensive provings of Lyssin collected by Dr. Hering for forty years, prepared from the crude saliva on the Hahnemannian plan and taken by the mouth. Though not inserted into the skin by inoculation, the symptoms obtained were so characteristic of hydrophobia that they point directly to action upon the nervous system. This effect could only be produced by means of the process of potentiation, by which the nosode is so modified as to preclude any chemical or toxical effect.

Dr. Galtier injected the saliva of a mad dog into the veins of ten sheep, and they all remained perfectly well. At the same time he placed the saliva of the same dog in contact with the nerves of ten other sheep, and they all died, with every symptom of rabies. After injecting the virus of rabies into the veins of sheep, however, they were found to be immune against rabies on subsequent experiments. This proves that the disease is produced by the contact of the virus with the nerves, and not by the direct introduction into the circulation. If in the latter case it makes the animal immune, it is owing to the contact with the nerve exposed in the inoculation. The *modus operandi* in bites of venomous reptiles is similar, and hence they are also amenable to cure by homœopathic potencies, as in Thorer's case of a viper-bite cured by a few pellets of Lachesis, 30th cent. These facts being familiar to the homœopathician, entitle him to treat such cases, presenting more or less fatal symptoms, according to the strict homœopathic method, using only such simple surgical means as the dressing of the wound requires, but not otherwise meddling with it locally. But the present time does not afford to the homœopathiciaan the opportunity to treat such cases, which at once rouse a panic with the relatives of the patient and their physicians. The wound is generally cauterized in the old manner, and narcotics are given; or the patient is carried at once to the Pasteur Institute. Similarly, it is the case with certain contagious diseases, and other diseases thought to be contagious. The tendency is to carry the patients out of the family into the hospital; to isolate them and subject them to the routine practice presently accepted in the surgical and allopathic schools. Of course, the patient and his relatives will have to be content with what is done with them.

Dante's inscription on the entrance into the Inferno is sometimes applicable to the gates of the hospital. A patient is carried to the hospital with the reluctant consent of the relatives, who are assured that there is no help but the operation for—*e. g.*, appendicitis. The relatives are told by the surgeon that he is able to cure the case, and, at any rate, the best for it will be done that is possible to do. The relatives are confident and submit. After a few days they want to see the patient and hear how he is getting on. But they are refused permission to see him, and sent away with the consolation that everything is done for him and he is all right. After a few more days the relatives become anxious, but they are not allowed admittance. A few days later they are requested to remove the corpse from the hospital. This does not happen in a small, out-of-the-way place, but in a big hospital with a great name. No wonder that the common people get shy of these beneficent institutions.

Appendicitis claims more victims than necessary, on account of the preconceived opinion that it is a surgical disease, and of the incapacity of surgery to treat it medically. The apprehension that this disease may turn out fatally if not attended to, causes the urgent recommendation of early operation. If there is nothing found to justify the apprehension, operation is of no great importance. The surgeon cuts down for all that, and sews up the wound again. Since this curious attachment to the big bowel is considered an unnecessary freak of nature, constituting, in the mind of the surgeon, a useless remnant of the evolution of man from the monkey and lower animals, he thinks nothing of cutting it out. The patient need not know anything about it. Nay, some enthusiasts excise the appendix where there is no cause for alarm and the individual is in perfect health, as a precautionary measure, in order to prevent him from falling a prey to the dangerous thing in the future. It is said that a surgeon, for this reason, cut out the appendixes of all his children.

How lax the reasoning of the surgeon sometimes may be is shown in a recent case. A young lady fell on the stoop going up to her house, and was laid up with a pain in the abdomen, distention from flatulency, great tenderness of the integuments, and rise of temperature and pulse. Her doctor was afraid of appendicitis, and saying so, advised to send for an expert in such matters. He came and examined the case and agreed with the doctor that it might be so. It would be safest to carry the patient immediately to the hospital for an operation. But the father objected and said he would rather have his daughter die from the disease in the house than submit her to an operation in a hospital. Then the surgeon withdrew his advice, ordered an inunction of the right iliac region with Iodine, and went away. The homœopathician was now called in who had treated the patient formerly. He could not find any trace of appendicitis, and treated her *lege artis homœopathicae* with good results. About a week after the two doctors saw the patient again and—*mirabile dictu*—gave the homœopathician credit for having cured the appendicitis, which certainly was also to their credit. This admonition of early operating must work a great deal of mischief, not only in this special affection, but also in others. Having no means to combat it medically, these surgeons do indeed the best they think of, though in point of fact it amounts to the worst; but, on the other hand, the avoidance of the operation, be it ever so successful, is of the greatest benefit to the patient, because it is really the best that could have been done, if the operation was not necessary.

The surgical bias is much to be deplored, because it prevents many cures in cases where operation promises no more certainty of a beneficial result than the medical—*i. e.*, homœopathic treatment. What has been predicated applies equally to other diseases, where the surgeons feel justified in interfering as long as they see a chance of success. This success must, however, always be doubtful when good results can only be reached in the non-surgical way. This bias is brought about and followed by their fallacious reasoning upon the appearances in operations during lifetime and *post-mortem* examinations. The pathological processes which present only the signs of disintegration of the tissues during the time of their elimination, shown by the decay and foul odors, are for them the indications of the severity of the disease, for which there is no help other than removal of the diseased part by the knife. But they do not understand that these processes are instituted by nature as remedial measures. The organism itself, in its wonderful construction, has enormous power and many auxiliaries for overcoming the inroads of miasms and violent lesions of its system, which, if aided by judicious homœopathic treatment, often recovers, to the surprise even of the physician familiar with the beautiful workings of nature. For nature—the life-force—must accomplish the cure. The surgeon, if necessary and successful, can only remove the impediments which the disease opposes to healing, but cannot cure.

The stricture of Dr. William P. Wesselhœft upon a paper presented to the International Hahnemannian Association at its last meeting by the Chairman of the Surgical Bureau, who counsels to "evacuate pus wherever it can be located, whether beneath the periosteum or in the chest, or in the abdomen or in the axilla" (*Proceedings I. H. A.*, 1896, p. 277), is well worth repeating: "We do not go for pus with a knife wherever we find it as Hahnemannians. We do not look upon pus as damnation or sure death if it is not evacuated. Many repeated experiences of others, as well as my own, have shown me that even large quantities of pus can be absorbed and are absorbed without the slightest danger to the patient."

In the admonitions of the surgeons to operate as early as possible is revealed a want of confidence in themselves, as they know they are not able to heal the disease while there is time, by medication on homœopathic principles. It is certainly impossible at that early time to diagnose the affection of the appendix depending upon the impaction of foreign material, and it would be inadmissible to presuppose this in every case and find only the surgical interference indicated. Clearly, at that time nothing else is indicated than the administration of the homœopathic remedy, and experience proves that many cases in this stage are actually healed. If, in the event of the case going on to suppuration, the operation is dictatorially demanded, because the organ is then in a decayed condition, even then it is doubtful whether nature has not means to prevent the fatal effects expected by the surgeon, under judicious Hahnemannian management, because such cases have also been reported cured.

There is another alleged source of diseases which of late years has taken hold of the surgical mind; the poisoning powers of the ptomaines, or substances which originate in the organism itself and produce diseases of their own. They are not amenable to surgery, and have to be met by appropriate medicine, as, *e. g.*, the typhoid condition of the system, depending apparently upon the degeneration of the Peyer's glands.

The new branch of surgery called orificial surgery pretends to cure remote symptoms of disease by operation upon the orifices of the body, which are deemed to be connected with the organs affected by the nerves, and a cure is attempted and excused by reflex action. Clearly, this is not homœopathic but allœopathic doctrine, which we have learned to fear in the treatment of the sick.

The greatest danger, of course, does not come from the surgeons of the highest rank, who know perfectly well what they are about, but from the beginners who are encouraged by the cry of their teachers: Operate! operate! and hence are apt to follow their advice. They do not care for medication in such cases, and much less for homœopathic medication, of which they know nothing. They are ambitious and want to make their mark by carving their way into the favor of the public with a scalpel. But "it costs lots of life to make a surgeon" (*Trans. Am. Inst.*, 1896, p. 383).

How essential for rational action in medicine is *correct thinking on the part of the physician.* How baleful are the consequences of introducing errors and fallacies into the practice—errors which must multiply with the daily needs of the suffering people. The above-quoted sentence of Hahnemann regarding the communication of disease, if carried out in strictness, would save a world of misery, not only to the patients, but also to the physicians themselves. Such errors as are committed in the neglect of the observations of wise men, come home to roost, and the physicians guilty of it have to deplore their losses and pains frequently in their own families and upon themselves. How do they torture their own children with their crude measures, though their own heart must bleed when they see how little they avail against their own ardent desire to help them!

How many breasts are sacrificed to the mania for operating upon any tumor or induration of the mammary gland! To take out one or the other breast is as nothing, and nobody questions the consequences. Most of these affections are amenable to internal medicinal treatment, and there is always the chance left that if those glands are not removed, they at least do not increase and incommode the patients so as to shorten their lives. Even the incurable cases have been benefited by homœopathic treatment, in rendering the pains less, and producing the only comfort possible when surgery is entirely out of question.

How many ovaries are excised which did not call for surgical interference at all, and are accessible to successful homœopathic treatment! The unsexing of women who want to shirk their matrimonial duty to bear children burdens them and the surgeons with a crime for which there is no excuse in the law-book of humanity. There would be more sense in unsexing the brutes who ruin a poor girl and afterward deny her reparation and a decent life. The Lothario goes free and his victim lives in shame, and often dies in misery.

Healthy eyes have been taken out for fear the morbid eye just enucleated might carry the affliction for which it was done to the sound eye. Oh, what barbarities are committed in the name of science which all these scientific men adore!

And yet it seems that those advocates of surgery in the most extended sense, and of material medication with the maximum dose which the system is calculated to bear, might be induced to mend their ways somewhat by applying a little logic to their practice. They see the material, visible part of the organism, but neglect the immaterial, invisible force which directs the physiological processes in the living body. They deny and ridicule the idea of a life-force such as Hahnemann conceived, which alone explains the action of medicines and teaches how to prepare and administer them. Though they bow down before the *vis medicatrix naturæ*, they do not see that thereby, unknowingly, they acknowledge the life-forces under another name, sanctified by age and authority. They should trust it a great deal more and learn to assist it by suitable medicines, such as Hahnemann has taught us to prepare—medicines which, escaping the coarse action of crude drugs, are elevated into healing forces coming from the great sources of all power in nature. Though all the virulent and inert substances in existence, on the earth and its surroundings, enter into the composition of the organism, it moves and lives in perfect harmony as the proper instrument for our rational spirit (*Organon*, § 9). Why, then, should not the life-force be capable of throwing the dangerous constituents back into their proper relation, when assisted by the well-adapted homœopathic medicine, corresponding in similarity with the nature of the life-force, in more or less high potency?

This the homœopathic surgeon at least should remember, and he should not cease to perfect himself as much in the practice of true homœopathics as in the skill of an operator, the proficiency in both of which, then, will make him a perfect physician.

*Ceterum censeo macrodosiam esse delendam.*

